# Nonmedical Use of Stimulants Is Associated With Riskier Sexual Practices and Other Forms of Impulsivity

**DOI:** 10.1097/ADM.0000000000000448

**Published:** 2018-11-02

**Authors:** Jon E. Grant, Sarah A. Redden, Katherine Lust, Samuel R. Chamberlain

**Affiliations:** Department of Psychiatry & Behavioral Neuroscience University of Chicago, Chicago, IL (JEG, SAR); Boynton Health Service, University of Minnesota, Minneapolis, MN (KL); Department of Psychiatry, University of Cambridge, and Cambridge and Peterborough NHS Foundation Trust, Cambridge, UK (SRC).

**Keywords:** addiction, amphetamine, impulsivity, methylphenidate

## Abstract

**Background:**

This study sought to examine the occurrence of the nonmedical use of prescription stimulants (amphetamines and methylphenidate) in a university sample and their associated physical and mental health correlates, including potential relationships with risky sexual practices.

**Methods:**

A 156-item anonymous online survey was distributed via e-mail to a sample of 9449 university students. Current use of alcohol and drugs, psychological and physical status, and academic performance were assessed, along with questionnaire-based measures of impulsivity and compulsivity.

**Results:**

A total of 3421 participants (59.7% female) were included in the analysis. 6.7% of the sample reported current/recent nonmedical use of prescription stimulants, while an additional 5.8% reported misuse in the past. Nonmedical use of prescription stimulants was associated with lower grade point averages, and with taking a broad range of other drugs (including alcohol, nicotine, illicit substances, and consumption of caffeinated soft drinks). Nonmedical use of stimulants was also significantly associated with impulsivity (Barratt scale), prior treatment for substance use problems, and elevated occurrence of disordered gambling, post-traumatic stress disorder, and anxiety; but not depression symptoms or binge-eating disorder (though it was associated with using drugs to lose weight). The relationship with probable attention-deficit/hyperactivity disorder (ADHD) on screening was not significant but was numerically elevated. Finally, those using nonmedical prescribed stimulants were significantly more sexually active (including at a younger age), and were less likely to use barrier contraception.

**Conclusions:**

Nonmedical use of prescription stimulants is common in young adults and has profound public health associations including with a profundity of other drug use (licit and illicit), certain mental health diagnoses (especially gambling, anxiety, and post-traumatic stress disorder ), worse scholastic performance, and riskier sexual practices. The majority of people with nonmedical use of prescription stimulants do not have ADHD, and its link with current ADHD symptoms was less marked than for certain other disorders. Clinicians should screen for the misuse of prescription stimulants as they may be associated with a range of problematic behaviors. Risk of diversion (which may be higher for those living in shared accommodation and those with substance use disorder history) merits careful assessment before prescribing stimulant medication.

Concerns about the nonmedical use of prescription stimulants (ie, surreptitious unsupervised use of amphetamines or methylphenidate originally prescribed for another) have been increasing and have been particularly focused on diversion and misuse of these medications by adolescents and young adults (Compton and Volkow, 2006; Poulin, 2007). The lifetime prevalence of nonmedical use of stimulants is common with a recent study (the National Epidemiologic Survey on Alcohol and Related Conditions, NESARC) reporting a rate of 4.7% in the United States (Huang et al., 2006). The corresponding rate of abuse and/or dependence on amphetamines was 2.0%, thereby reflecting the fact that far more people misuse amphetamines than meet criteria for full substance use disorder.

The nonmedical use of stimulants has been well documented in university students around the world (Kroutil et al., 2006; McNiel et al., 2011; Maier et al., 2013; Majori et al., 2017). A study of 390 university students found that 7.5% reported nonmedical use of prescription stimulants within the past 30 days; 60% reported knowing students who misused stimulants; and 50% agreed or strongly agreed that prescription stimulants were “easy to get on this campus” (Weyandt et al., 2009). Other studies have found that that 6.0% of high school seniors and 6.7% of college students have misused stimulants (McCabe et al., 2006, 2007). A large review of 21 studies found that past year nonprescribed stimulant use ranged from 5% to 35% in college-age individuals and that white Caucasian individuals, members of fraternities and sororities, individuals with lower grade point averages, and individuals who reported attention-deficit/hyperactivity disorder (ADHD) symptoms were at highest risk of misusing stimulants (Wilens et al., 2008). A study of 1153 undergraduates was conducted comparing those with stimulant prescriptions who use them appropriately, those who misuse their prescription stimulants, those who obtain and use stimulants without a prescription, and those who do not use stimulant medications at all (Hartung et al., 2013). This study found that both the students who abused their prescriptions and those who obtained stimulants illegally reported problems with other substance use, as well as insomnia and restlessness.

The relatively high rates of stimulant misuse among university students have been attributed to various factors such as the desire for a “cognitive enhancer” to help with school work, to lose weight and look better physically, to improve alertness, the positive portrayal of stimulants on the Internet, and perception that stimulants assist in coping with psychological distress (Poulin, 2007; Schepis and Krishnan-Sarin, 2008; Wilens et al., 2008; Weyandt et al., 2009; Ford and Ong, 2014; Looby and Sant’Ana, 2018). One study of college students found that 28.6% agreed or strongly agreed that the nonmedical use of prescription stimulants could help earn higher grades (Arria et al., 2018). For a review of the multi-faceted reasons for stimulant use on college campuses, see Bavarian et al. (2015).

Despite this high prevalence, relatively little is known about the associations between nonmedical stimulant use, academic performance, socialization, and self-esteem in university settings. Therefore, this study sought to examine both the occurrence of nonmedical use of stimulants (amphetamines and methylphenidate) in a university sample, and the associated emotional and functional consequences of misuse. Based on the previous literature (Wilens et al., 2008; Blanco et al., 2013), we hypothesized that the nonmedical use of stimulants would be associated with poor self-esteem and impairments in academic performance; higher rates of other substance problems, depression and anxiety; and elevated questionnaire-measures of impulsivity including personality and sex-related behaviours.

## METHODS

### Survey Design

The Department of Psychiatry and Behavioral Neuroscience at the University of Chicago and Boynton Health at the University of Minnesota jointly developed the *Health and Addictive Behaviors Survey* to assess mental health and well-being in a large sample of university students. The survey included basic demographics as well as questions from a number of validated screening tools examining mental health and psychological well-being. All study procedures were carried out in accordance with the Declaration of Helsinki and were approved by the institutional review board of the University of Minnesota.

### Participants

A subsample of 10,000 college and graduate students at a large, nondenominational, and coeducational Midwestern university were chosen by random, computer-generated selection, from a total pool of approximately 60,000 students at the university. The survey was distributed over a 3-week period during fall semester, with invitations being sent via e-mail, and surveys being completed online. Of the 10,000 e-mail invitations, 9449 were successfully received by the recipients (ie, did not bounce back). Of the 9449 students with valid e-mails who received the e-mail invitation, 3659 (38.7%) responded to a majority of the questions—examples 99.2% responded to the question about their class status; 91.9% responded to the gender question. This response rate is commensurate with other national or university health surveys (Baruch, 1999; Cook et al., 2000; Baruch and Holtom, 2008; Van Horn et al., 2009; Odlaug et al., 2013). The analysis for this paper was based on those that responded to the prescription stimulant question 3421/3659 = 93.5%.

Recipients of the e-mail were first required to view the institutional review board-approved online informed consent page, at which point students could choose to participate in the survey or opt out. The survey asserted that all information was both anonymous and confidential. Compensation was offered at the conclusion of the survey by randomly selecting respondents to receive tablet computers (3 winners) or gift certificates to an online retailer in the amounts of $250 (4 winners), $500 (2 winners), and $1000 (1 winner). Participants were required to review all survey questions to be eligible for prize drawings, but were not required to answer all questions, due to the sensitive nature of some of them.

### Assessments

The self-report survey consisted of 156 questions and took participants approximately 30 minutes to complete. Survey questions assessed demographic information, sexual behavior, self-reported academic achievement (ie, grade point average [GPA]), and clinical characteristics, including mental health and substance use issues.

In terms of the nonmedical use of stimulants, participants were asked “Please mark the frequency with which you have used prescription stimulants or amphetamines (e.g. Ritalin, Adderall, Concerta) within the past 12 months. DO NOT include drugs prescribed for you.” Frequency options were as follows: never, occasionally, daily, or used in past but have not used within past 12 months.

Participants also completed the following measures:

***Alcohol Use Disorders Identification Test (AUDIT)***. The AUDIT is a well-validated, 10-item questionnaire used to assess alcohol use behaviors and related problems (Saunders et al., 1993). A score of 8 or greater indicates hazardous or harmful alcohol use.
***Drug Abuse Screening Test (DAST-10)***. The DAST is a 10-item, yes/no measure of problematic substance use. A score of 3 is used to screen for a drug use disorder (Skinner, 1982; Yudko et al., 2007).
***Patient Health Questionnaire (PHQ-9)***. The PHQ-9 is a 9-item measure of depressive symptoms based directly on DSM-IV-TR criteria for major depressive disorder (Kroenke et al., 2001).
***Generalized Anxiety Disorder 7 (GAD-7)***. The GAD-7 is a 7-item, screening tool for generalized anxiety disorder (GAD) (Spitzer et al., 2006). Total scores of 10 or greater indicate clinically significant anxiety.
***Adult ADHD Self-Report Scale (ASRS-v1.1)***. The ASRS is a 6-item screening tool for ADHD (Kessler et al., 2005).
***Minnesota Impulsive Disorders Interview (MIDI).*** The MIDI is a screening instrument for impulse control disorders, including binge eating disorder and gambling disorder (Grant, 2008).
***Barratt Impulsiveness Scale, Version 11 (BIS-11)***. The BIS-11 is a 30-item measure designed to assess impulsivity across 3 dimensions: attentional (inability to concentrate), motor (acting without thinking), and non-planning (lack of future orientation) (Stanford et al., 2009).

### Data Analysis

Only respondents who answered the question regarding the nonmedical use of stimulants were included in the analyses (N = 3421). Participants were grouped into the following categories: those who never used such stimulants, those who had used stimulants in the previous 12 months, and those who had used in the past but not in the past 12 months. Significant main effects of group were identified using likelihood ratios or χ^2^ tests as indicated in the text. SPSS was used for all statistical analyses (version 24; IBM Corp). Statistical significance was defined as *P* ≤ 0.05, 2-tailed, Bonferroni corrected for the number of measures in each Table of interest (i.e. for each category of variable). Pairwise comparison (group 1 vs group 2; group 1 vs group 3; group 2 vs group 3) were performed when the overall effect of group was significant for a given measure.

Missing data were missing completely at random (MCAR) and the analysis was conducted using listwise deletion. Missing values were analyzed using Little's MCAR. We included all quantitative variables and 5 categorical demographic variables to test for missing completely at random. The results came back as χ^2^ = 34.295, df = 34, significance = 0.454, which indicates missing completely at random. By far the most common approach to the missing data is to simply omit those cases with the missing data and analyze the remaining data. This approach is known as the complete case (or available case) analysis or listwise deletion. Listwise deletion is the most frequently used method in handling missing data. Although this may introduce bias in the estimation of the parameters, if the assumption of MCAR is satisfied, a listwise deletion is known to produce unbiased estimates and conservative results. Also, because this was a large sample, where power was not an issue, the assumption of MCAR was satisfied and listwise deletion seemed reasonable.

## RESULTS

Of the 3421 participants, 230 (6.7%) reported current or recent nonmedical use of prescription stimulants, while an additional 199 (5.8%) reported nonmedical use in the past (>1 year ago). The demographic variables for the entire sample are presented in Table 1. Those who reported nonmedical use of prescription stimulants were more likely to be undergraduates, had lower GPAs, and were more likely to live in fraternity/sorority houses. There was no statistical difference based on gender (χ^2^ = 6.498, df = 12, *P* = 0.889).

**TABLE 1:** Demographics of University Students Based on Nonmedical Use of Prescription Stimulants

Variable	Students Who Currently Misuse Prescription Stimulants (n = 230)	Students Who Have Misused Prescription Stimulants in the Past (n = 199)	Students Who Have Never Misused Prescription Stimulants (n = 984)	Statistic	Raw *P* Value
Sex, female, n (%)	135 (60.3)	110 (57.6)	574 (61.0)	χ^2^ (4) = 2.75LR	0.601
Year in college, n (%)					
Undergraduate	189 (82.2)^ab^	124 (62.3)^a^	669 (68.0)^b^	χ^2^ (4) = 28.84LR	<0.001^*^
Graduate	41 (17.8)	73 (36.7)	314 (31.9)		
Non-degree	0 (0.0)	2 (1.0)	1 (0.1)		
Full time student, n (%)	208 (90.4)	178 (89.4)	916 (93.1)	χ^2^ (2) = 3.96LR	0.126
GPA				χ^2^ (2) = 16.23LR	<0.001^*^
<3.00	45 (19.7)^a^	30 (15.1)	98 (10.0)^a^		
3.00 or higher	184 (80.3)	169 (84.9)	879 (90.0)		
Involved in a fraternity or sorority, n (%) YES	41 (17.9)	18 (9.0)	131 (13.3)	χ^2^ (2) = 7.29LR	0.026
Living arrangements, n (%)					
Residence hall	31 (13.6)^ab^	18 (9.0)^a^	158 (16.1)^b^	χ^2^ (4) = 21.84	<0.001^*^
Fraternity or sorority house	16 (7.0)	3 (1.5)	24 (2.4)		
Off campus	181 (79.4)	178 (89.4)	802 (81.5)		

All numbers are % (N) unless otherwise stated. LR denotes results based on Likelihood Ratio otherwise results based on Pearson χ^2^.

^*^P < 0.05 significant with Bonferroni correction (threshold *P* < 0.0083). Post hoc analysis corrected for alpha inflation using 0.05/3 = *P* < 0.017. Superscript letters designate where there is a statistical difference between groups. If all 3 groups have an “a” then they all are statistically different from each other (group 1 vs Group 2) (group 1 vs group 3) and (group 2 vs group 3). If there is a series say “a”, “b,” “ab” then group 1 is different from group 3; Group 2 is different from Group 3 but group 1 is not different then group 2.

Alcohol and drug use by the participants is presented in Table 2. Nonmedical use of prescription stimulants was significantly associated with more alcohol and nicotine problems, as well as the use of a wide range of drugs.

**TABLE 2:** Alcohol, Tobacco, and Illicit Drug Use in Students Based on Nonmedical Use of Prescription Stimulants

Variable	Students Who Currently Misuse Prescription Stimulants	Students Who Have Misused Prescription Stimulants in the Past	Students Who Have Never Misused Prescription Stimulants	Statistic	Raw *P* Value
Age at first use of cigarettes or nicotine					
Never used	33 (14.3)^a^	37 (18.6)^b^	404 (41.1)^ab^	χ^2^ (6) = 133.38	<0.001^*^
<14 years	33 (14.3)	34 (17.1)	73 (7.4)		
15–17 years	99 (43.0)	80 (40.2)	196 (19.9)		
18 years or older	65 (28.3)	48 (24.1)	311 (31.6)		
Frequency of e-cigarette use					
Never	52 (26.4)^ab^	81 (50.0)^a^	315 (54.3)^b^	χ^2^ (8) = 61.12	<0.001^*^
Not within past year	48 (24.4)	44 (27.2)	124 (21.4)		
Rarely	66 (33.5)	25 (15.4)	98 (16.9)		
Occasionally	19 (9.6)	8 (4.9)	30 (5.2)		
Daily	12 (6.1)	4 (2.5)	13 (2.2)		
Frequency of alcohol consumption					
Never	3 (1.3)^ab^	11 (5.5)^a^	32 (3.3)^b^	χ^2^ (8) = 42.02	<0.001^*^
Monthly or less	14 (6.1)	24 (12.1)	130 (13.2)		
2–4 times a month	69 (30.0)	65 (32.7)	387 (39.3)		
2–3 times a week	97 (42.2)	71 (35.7)	342 (34.8)		
4+ times a week	47 (20.4)	28 (14.1)	93 (9.5)		
AUDIT Total					
Score <8	67 (29.1)^a^	97 (49.0)^a^	633 (64.4)^a^	χ^2^ (2) = 99.56	<0.001^*^
Score 8 or higher	163 (70.9)	101 (51.0)	350 (35.6)		
Non-prescription amphetamines					
Never	200 (87.3)^a^	174 (88.3)^a^	956 (97.3)^a^	χ^2^ (8) = 76.39LR	<0.001^*^
In past, not within past 12 months	9 (3.9)	21 (10.7)	15 (1.5)		
Rarely	10 (4.4)	2 (1.0)	10 (1.0)		
Occasionally	7 (3.1)	0 (0.0)	0 (0.0)		
Daily	3 (1.3)	0 (0.0)	2 (0.2)		
Cocaine					
Never	120 (53.1)^a^	118 (60.2)^a^	888 (91.3)^a^	χ^2^ (6) = 264.57LR	<0.001^*^
In past, not within past 12 months	39 (17.3)	65 (33.2)	55 (5.7)		
Rarely	50 (22.1)	11 (5.6)	28 (2.9)		
Occasionally	17 (7.5)	2 (1.0)	2 (0.2)		
Daily	0 (0.0)	0 (0.0)	0 (0.0)		
Opiates (eg, heroin, opium)					
Never	213 (93.0)^a^	179 (89.9)^b^	965 (98.5)^ab^	χ^2^ (8) = 45.41LR	<0.001^*^
In past, not within past 12 months	8 (3.5)	17 (8.5)	12 (1.2)		
Rarely	3 (1.3)	2 (1.0)	1 (0.1)		
Occasionally	2 (0.9)	0 (0.0)	1 (0.1)		
Daily	3 (1.3)	1 (0.5)	1 (0.1)		
Inhalants					
Never	209 (91.7)^a^	181 (92.3)^b^	960 (98.2)^ab^	χ^2^ (6) = 32.09LR	<0.001^*^
In past, not within past 12 months	13 (5.7)	13 (6.6)	13 (1.3)		
Rarely	5 (2.2)	2 (1.0)	3 (0.3)		
Occasionally	2 (0.2)	0 (0.0)	1 (0.1)		
Daily	0 (0.0)	0 (0.0)	0 (0.0)		
Hallucinogens					
Never	101 (44.1)^a^	95 (48.5)^a^	820 (83.6)^a^	χ^2^ (8) = 241.03LR	<0.001^*^
In past, not within past 12 months	47 (20.5)	76 (38.8)	101 (10.3)		
Rarely	55 (24.0)	15 (7.7)	44 (4.5)		
Occasionally	24 (10.5)	10 (5.1)	16 (1.6)		
Daily	0 (0.0)	0 (0.0)	2 (0.9)		
Marijuana					
Never	7 (3.0)^a^	4 (2.0)^a^	34 (3.5)^a^	χ^2^ (8) = 125.98	<0.001^*^
In past, not within past 12 months	30 (13.0)	59 (29.8)	291 (29.6)		
Rarely	45 (19.6)	59 (29.8)	367 (37.3)		
Occasionally	96 (41.7)	50 (25.3)	240 (24.4)		
Daily	52 (22.6)	26 (13.1)	51 (5.2)		
Prescription pain medication					
Never	124 (54.1)^a^	123 (62.1)^a^	897 (91.6)^a^	χ^2^ (8) = 251.72LR	<0.001^*^
In past, not within past 12 months	54 (23.6)	68 (34.3)	63 (6.4)		
Rarely	37 (16.2)	6 (3.0)	17 (1.7)		
Occasionally	9 (3.9)	1 (0.5)	2 (0.2)		
Daily	5 (2.2)	0 (0.0)	0 (0.0)		
Sedatives					
Never	154 (67.5)^a^	153 (76.9)^a^	937 (95.5)^a^	χ^2^ (8) = 167.19LR	<0.001^*^
In past, not within past 12 months	33 (14.5)	35 (17.6)	23 (2.3)		
Rarely	26 (11.4)	4 (2.0)	12 (1.2)		
Occasionally	12 (5.3)	6 (3.0)	6 (0.6)		
Daily	3 (1.3)	1 (0.5)	3 (0.3)		

LR denotes results based on Likelihood Ratio otherwise results based on Pearson χ^2^. All values are n (%) unless otherwise stated; AUDIT, Alcohol Use Disorders Identification Test.

^*^P **<** 0.05 significant with Bonferroni correction (threshold *P* < 0.0042). Post hoc analysis corrected for alpha inflation using 0.05/3 = *P* < 0.017. If all 3 groups have an “a” then they all are statistically different from each other (group 1 vs Group 2) (group 1 vs group 3) and (group 2 vs group 3). If there is a series say “a”, “b,” “ab” then group 1 is different from group 3; Group 2 is different from Group 3 but group 1 is not different then group 2.

The sexual behavior of students based on their misuse of prescription stimulants is presented in Table 3. Students who reported nonmedical use of prescription stimulants were significantly more likely to be sexually active, sexually active at a younger age, and to be sexually active without using some form of barrier contraceptive method.

**TABLE 3:** Sexual Behavior in University Students Based on Nonmedical Use of Prescription Stimulants

Variable	Students Who Currently Misuse Prescription Stimulants	Students Who Have Misused Prescription Stimulants in the Past	Students Who Have Never Misused Prescription Stimulants	Statistic	Raw *P* Value
Has been sexually active					
Yes	216 (93.9)^a^	189 (95.5)^b^	852 (87.3)^ab^	χ^2^ (2) = 17.33	<0.001^*^
No	14 (6.1)	9 (4.5)	124 (12.7)		
Age at first sexual activity with another					
<11 years	2 (0.9)^a^	5 (2.6)^b^	6 (0.7)^ab^	χ^2^ (8) = 92.88LR	<0.001^*^
12–14 years	33 (15.3)	35 (18.5)	33 (3.9)		
15–17 years	123 (56.9)	94 (49.7)	402 (47.2)		
18–20 years	54 (25.0)	46 (24.3)	352 (41.3)		
21 years or older	4 (1.9)	9 (4.8)	59 (6.9)		
Frequency of physical barrier use					
<50% of the time	104 (48.1)^a^	89 (47.1)^b^	314 (36.9)^ab^	χ^2^ (6) = 19.79	0.003^*^
50–75% of the time	26 (12.0)	22 (11.6)	98 (11.5)		
76–95% of the time	37 (17.1)	39 (20.6)	161 (18.9)		
96–100% of the time	49 (22.7)	39 (20.6)	277 (32.6)		

LR denotes results based on Likelihood Ratio otherwise results based on Pearson χ^2^. All values are n (%) unless otherwise stated.

^*^P **<** 0.05 significant with Bonferroni correction (threshold *P* < 0.017). Post hoc analysis corrected for alpha inflation using 0.05/3 = *P* < 0.017. If all 3 groups have an “a” then they all are statistically different from each other (group 1 vs group 2) (group 1 vs group 3) and (group 2 vs group 3). If there is a series say “a”, “b,” “ab” then group 1 is different from group 3; group 2 is different from Group 3 but group 1 is not different then group 2.

The health and mental health aspects of the participants are presented in Table 4. Nonmedical use of prescription stimulants was significantly associated with greater intake of caffeinated soft drinks, more impulsivity on the Barratt scale, history of treatment for drug/alcohol use disorder, using drugs to lose weight, higher rates of gambling disorder, post-traumatic stress disorder (PTSD), and worse anxiety symptoms. Nonmedical use of prescription stimulants was not significantly associated with binge-eating disorder, treatment for psychological/emotional problems, or taking prescribed medication; furthermore, the group differences positive screen for ADHD symptoms was not significant after Bonferroni correction.

**TABLE 4:** Impulsive Behaviors and Psychiatric History of University Students Based on Nonmedical Use of Prescription Stimulants

Variable	Students Who Currently Misuse Prescription Stimulants	Students Who Have Misused Prescription Stimulants in the Past	Students Who Have Never Misused Prescription Stimulants	Statistic	Raw *P* Value
Amount of caffeinated soft drinks consumed over the past week n (%)					
Never	93 (40.8)	76 (38.4)^a^	489 (50.5)^a^	χ^2^ (10) = 28.75LR	0.001^*^
1–2 drinks	82 (36.0)	75 (37.9)	303 (31.3)		
3–6 drinks	35 (15.4)	23 (11.6)	114 (11.8)		
7–12 drinks	6 (2.6)	19 (9.6)	39 (4.0)		
13–23 drinks	7 (3.1)	2 (1.0)	13 (1.3)		
24 or more drinks	5 (2.2)	3 (1.5)	10 (1.0)		
BIS Total Mean (SD)	66.09 (10.29)^ab^	63.13 (9.94)^a^	61.08 (9.97)^b^	F (2,1301) = 22.657	<0.001^*^
Gambles				χ^2^ (2) = 14.06	
Yes	41 (18.2)^a^	28 (14.4)	93 (9.7)^a^		0.001^*^
Gambling disorder?				χ^2^ (2) = 12.56LR	
Positive screen	6 (2.7)^a^	0 (0.0)	3 (0.3)^a^		0.002^*^
Binge eating disorder?					
Positive screen	8 (3.6)	5 (2.6)	34 (3.6)	χ^2^ (2) = 0.49	0.782
Has been treated for drug/alcohol use disorder				χ^2^ (2) = 30.52	
Yes	12 (5.3)^a^	18 (9.2)^b^	17 (1.8)^ab^		<0.001^*^
Has been treated for psychological/emotional problems					
Yes	99 (43.6)	86 (44.1)	350 (36.2)	χ^2^ (2) = 7.18	0.028
Currently taking prescribed mental health medication (s)					
Yes	53 (23.3)	41 (20.9)	173 (17.9)	χ^2^ (2) = 3.92	0.141
Has used drugs in order to lose weight					
Yes	36 (28.1)^a^	19 (18.6)^b^	34 (7.0)^ab^	χ^2^ (2) = 45.75	<0.001^*^
PHQ-9 Total					
Score of less than 10	205 (92.8)	179 (94.2)	901 (94.8)	χ^2^ (2) = 1.49	0.474
Score of 10 or more	16 (7.2)	11 (5.8)	49 (5.2)		
PTSD				χ^2^ (2) = 12.57	
Positive screen	57 (25.7)^a^	37 (19.2)	150 (15.7)^a^		0.002^*^
GAD-7 Total Mean (SD)	6.04 (5.05)	2.19 (5.28)	5.23 (5.14)	F (2,1350) = 4.252	0.014
Anxiety total					
Grouped	100 (45.5)^a^	90 (46.6)	545 (58.0)^a^	χ^2^ (6) = 21.62	0.001^*^
No Anxiety (score 0–4)	67 (30.5)	61 (31.6)	223 (23.7)		
Mild (score 5–9)	38 (17.3)	23 (11.9)	99 (10.5)		
Moderate (score 10–14)	15 (6.8)	19 (9.8)	73 (7.8)		
Severe (score 15–21)					
ADHD					
Positive screen	59 (26.7)	48 (24.9)	172 (18.2)	χ^2^ (2) = 10.58	0.005

LR denotes results based on Likelihood Ratio otherwise results based on Pearson χ^2^. All values are n (%) unless otherwise stated; BIS, Barratt Impulsiveness Scale, Version 11; PHQ-9 = Patient Health Questionnaire.

^*^P **<** 0.05 significant with Bonferroni correction (threshold *P* < 0.0038). Post hoc analysis corrected for alpha inflation using 0.05/3 = *P* < 0.017. If all 3 groups have an “a” then they all are statistically different from each other (group 1 vs group 2) (group 1 vs group 3) and (group 2 vs group 3). If there is a series say “a”, “b,” “ab” then group 1 is different from group 3; group 2 is different from group 3 but group 1 is not different then group 2.

## DISCUSSION

This study examined the nonmedical use of stimulants in a large sample of university students (ie, use of stimulants prescribed for another person); and ways in which stimulant misuse was related to a range of demographic/clinical measures, and questionnaire-based measures of impulsivity. We found that 6.7% of the sample reported current/recent nonmedical use of prescription stimulants. The rate of nonmedical use of stimulants was somewhat higher than the rate in the population at large (Huang et al., 2006) but consistent with several published university samples (Weyandt et al., 2009; Maier et al., 2013). Rates seem to be affected by the nature of the sample and the definitions deployed. Because our sample comprised university students, the use of stimulants here may be understandably higher than in (typically older) community cohorts. Young adulthood is a time when scholarly performance assumes significant meaning for many and so a motivation to maximize performance may drive the nonmedical use of stimulants. Unfortunately, early adulthood is also an important time when addictive symptoms may develop, which may then have negative effects during later adulthood as this period is often critical for forming close relationships, scholastic achievements, and developing one's career. Although most of our survey respondents were female (a percentage consistent with the overall rate of females in United States universities according to the National Center for Education Statistics [https://nces.ed.gov/fastfacts/display.asp?id=372]), we found that prescription stimulant misuse was no higher in males then in females.

In terms of demographic measures, our results are in keeping with a large literature on the subject (Poulin, 2007; Schepis and Krishnan-Sarin, 2008; Wilens et al., 2008; Weyandt et al., 2009; Hartung et al., 2013; Ford and Ong, 2014; Looby and Sant’Ana, 2018). Whether or not individuals are using nonmedical stimulants with the aim of improving their scholastic performance, these data suggest that this is an unsuccessful strategy. Misuse of prescription stimulants has been reported to be more common in competitive settings (McCabe et al., 2005), including when under performance pressure (Maier et al., 2013; Liakoni et al., 2015); and in individuals with higher family incomes (Teter et al., 2003). From a public health perspective, we observed a strong association between nonmedical use of prescription stimulant and earlier first sexual experience, greater likelihood of being currently sexually active, and lower use of barrier contraception. Nonmedical use of stimulants (and other substances) is an established risk factor for sexually transmitted infections (Degenhardt et al., 2014), and the current data identify several relevant associations, including with riskier sexual practices, and earlier sexual practices.

In addition to the commonly reported association between the nonmedical use of stimulants and other substance use, this study further found that there was an association with other problematic behaviors such as caffeine use, sexual behavior, and gambling. Taken together, these findings suggest a more global impulsive nature in young adults who misuse stimulants (evidenced in part by higher BIS-11 scores also observed). Whether stimulant misuse results in these other unhealthy behaviors (eg, amphetamine use make a person gamble more or use cocaine), is itself a result of 1 or more of these other behaviors (eg, disinhibition from alcohol or cocaine results in the nonmedical use of prescription ampehatmines), or is driven by a deeper underlying cognitive/physiological mechanism (eg, impulsivity as seen on the BIS-11) cannot be determined given the cross-sectional nature of these data. Cognitive, personality, and imaging research is needed to better understand these associations.

In terms of other mental health problems in the sample (beyond substance use), the nonmedical use of stimulants was significantly associated with symptoms of PTSD, and with anxiety (GAD-7); while an association was also seen with current ADHD symptoms, this was weaker and was not significant with Bonferroni correction. Relationships between stimulant misuse and depression (PHQ-9) and binge-eating disorder were not significant in this study. This is contrary to our expectations and could be due to limited power. For example, binge-eating disorder was uncommon and so group differences on its occurrence rate would have been hard to detect. Nonetheless, our data suggest that nonmedical use of stimulants is more strongly related to polysubstance use, anxiety and PTSD, than to ADHD and depressive symptoms, a view that may be quite different to the public (and perhaps clinical) perception that such individuals may be “self-medicating” for ADHD.

This study into the nonmedical use of stimulants has the advantage of being relatively large. Nonetheless, there are several limitations that should be noted. The study was cross-sectional and hence the direction of causality of any effects cannot be established—this would require longitudinal designs. There are limitations inherent in the study design—diagnostic assessment may be less accurate (more “noisy”) via such an online survey compared to in-person assessment by a clinician; there may be responder biases; and there may be under-reporting (though this possibility is mitigated by virtue of the survey being anonymous) (for an analysis of the complex relationship between anonymity and reporting stimulant use, please see Zander et al., 2016). Additionally, self-report questions pertaining to substance use have their own limitations: for example, individuals may not disclose the full extent of their use or may not report it accurately due to bias. Because this was an anonymous survey, we were not able to present the demographic characteristics of all individuals who were contacted, or to compare survey responders and non-responders in terms of their demographic characteristics. Furthermore, groups differed on some demographic characteristics and our data were reported without control for these differences. The study also did not assess medical misuse (ie, use of stimulant by the person it is prescribed for, but in ways not intended by the prescriber), and this is an important area for further research on this topic. Finally, we did not collect information about the particular chemical formulations of stimulants being taken by a given individual (eg, methylphenidate, dexamphetamine, and so forth).

In summary, this study found nonmedical use of prescription stimulants to be relatively common in young people, and such use was associated with a host of important public health concerns including with higher use of other substances (licit and illicit), anxiety and PTSD symptoms, and higher risk sexual practices (earlier age at first sexual experience and lower use of barrier contraception). Nonmedical use of prescription stimulants was linked with lower grade point average and only weakly (not significantly) with current ADHD symptoms, suggesting that using such drugs with a view to improving grades or ‘self-medicating’ ADHD out of clinical settings is not only unlikely to be helpful, but rather is likely to be harmful.
